# Cortical Bone Morphological and Trabecular Bone Microarchitectural Changes in the Mandible and Femoral Neck of Ovariectomized Rats

**DOI:** 10.1371/journal.pone.0154367

**Published:** 2016-04-29

**Authors:** Pei-Yu Hsu, Ming-Tzu Tsai, Shun-Ping Wang, Ying-Ju Chen, Jay Wu, Jui-Ting Hsu

**Affiliations:** 1 Department of Biomedical Imaging and Radiological Science, China Medical University, Taichung, 404, Taiwan; 2 Department of Biomedical Engineering, Hungkuang University, Taichung, Taiwan, 433, ROC; 3 Department of Orthopaedics, Taichung Veterans General Hospital, Taichung, 407, Taiwan; 4 Department of Food and Nutrition, Providence University, Taichung, 433, Taiwan; 5 Department of Biomedical Imaging and Radiological Sciences, National Yang-Ming University, Taipei, Taiwan, 112, ROC; 6 School of Dentistry, College of Medicine, China Medical University, Taichung, 404, Taiwan; 7 Department of Bioinformatics and Medical Engineering, Asia University. Taichung, 413, Taiwan; UAMS, UNITED STATES

## Abstract

**Objective:**

This study used microcomputed tomography (micro-CT) to evaluate the effects of ovariectomy on the trabecular bone microarchitecture and cortical bone morphology in the femoral neck and mandible of female rats.

**Materials and Methods:**

Twelve female Wister rats were divided into two groups: the control and ovariectomized groups. The rats in the ovariectomized group received ovariectomy at 8 weeks of age; all the rats were sacrificed at 20 weeks of age, and their mandibles and femurs were removed and scanned using micro-CT. Four microstructural trabecular bone parameters were measured for the region below the first mandibular molar and the femoral neck region: bone volume fraction (BV/TV), trabecular thickness (TbTh), trabecular separation (TbSp), and trabecular number (TbN). In addition, four cortical bone parameters were measured for the femoral neck region: total cross-sectional area (TtAr), cortical area (CtAr), cortical bone area fraction (CtAr/TtAr), and cortical thickness (CtTh). The CtTh at the masseteric ridge was used to assess the cortical bone morphology in the mandible. The trabecular bone microarchitecture and cortical bone morphology in the femoral necks and mandibles of the control group were compared with those of the ovariectomized group. Furthermore, Spearman’s correlation (*r*_*s*_) was conducted to analyze the correlation between the osteoporosis conditions of the mandible and femoral neck.

**Results:**

Regarding the trabecular bone microarchitectural parameters, the BV/TV of the trabecular bone microarchitecture in the femoral necks of the control group (61.199±11.288%, median ± interquartile range) was significantly greater than that of the ovariectomized group (40.329±5.153%). Similarly, the BV/TV of the trabecular bone microarchitecture in the mandibles of the control group (51.704±6.253%) was significantly greater than that of the ovariectomized group (38.486±9.111%). Furthermore, the TbSp of the femoral necks in the ovariectomized group (0.185±0.066 mm) was significantly greater than that in the control group (0.130±0.026mm). Similarly, the TbSp of the mandibles in the ovariectomized group (0.322±0.047mm) was significantly greater than that in the control group (0.285±0.041mm). However, the TbTh and TbN trends for the mandibles and femoral necks were inconsistent between the control and ovariectomized groups. Regarding the cortical bone morphology parameters, the TtAr of the femoral necks in the ovariectomized group was significantly smaller than that in the control group. There was no significant difference in the TtAr, CtAr, or CtTh of the femoral necks between the control and ovariectomized groups, and no significant difference in the CtTh of the mandibles between the control and ovariectomized groups. Moreover, the BV/TV and TbSp of the mandibles were highly correlated with those of the femurs (*r*_*s*_ = 0.874 and *r*_*s*_ = 0.755 for BV/TV and TbSp, respectively). Nevertheless, the TbTh, TbN, and CtTh of the mandibles were not correlated with those of the femoral necks.

**Conclusion:**

After the rats were ovariectomized, osteoporosis of the trabecular bone microarchitecture occurred in their femurs and mandibles; however, ovariectomy did not influence the cortical bone morphology. In addition, the parametric values of the trabecular bone microarchitecture in the femoral necks were highly correlated with those of the trabecular bone microarchitecture in the mandibles.

## Introduction

Osteoporosis is one of the most common major chronic diseases in humans (particularly menopausal women). According to the World Health Organization (WHO), osteoporosis is the second most common disease (after cardiovascular disease) [1]. Hundreds of millions of people worldwide experience osteoporosis [2]. In the United States and the European Union, one-third of menopausal women experienced osteoporosis [3]. In 1993, the WHO defined osteoporosis as a systemic bone disease, which is characterized by osteopenia, deterioration of bone tissue microstructure, brittle bones, and increased fracture risk [4]. According to the WHO diagnosis criteria, osteoporosis is defined as a bone mineral density (BMD) that lies 2.5 standard deviations or more below the mean value for young healthy female (T-score ≦ -2.5), [5]. Clinically, two types of osteoporosis exist: primary osteoporosis and secondary osteoporosis [6]. Regarding primary osteoporosis, type I primary osteoporosis is postmenopausal osteoporosis, which occurs when the amount of estrogen secretion and the speed of bone formation decrease, thereby increasing the speed of bone loss. Type II primary osteoporosis is senile osteoporosis, which occurs when the synthesis of active vitamin D decreases in elderly people, thus decreasing the gastrointestinal absorption of calcium, the activity of bone cells, and the amount of bone formation. Secondary osteoporosis is induced by certain diseases or drugs and occurs at any age in both males and females. Bone fracture resulting from osteoporosis (particularly hip fracture, vertebral compression deformation, and radial bone fracture [7]) can harm patients’ health and impair their ability to live independently. In addition, hip fracture and vertebral fracture can increase mortality in osteoporosis [8].

Both men and women may develop osteoporosis. However, women are more likely to develop osteoporosis than men because of the smaller skeletons and lower bone mass of women compared with those of men, and because of decreased estrogen secretion in menopausal women (i.e., estrogen deficiency can accelerate bone loss) [9, 10]. According to previous studies, women aged more than 50 years are more likely to experience bone fracture resulting from osteoporosis than men [11]. Multiple studies have examined ovariectomized rats to determine osteoporosis in menopausal women [12–17]. The predilection sites of bone fracture resulting from osteoporosis include the hip, vertebral bone, ribs, and wrist. Particularly, hip fracture resulting from osteoporosis occurs most frequently [18], and 14% to 58% of patients who experience hip fracture resulting from osteoporosis die within 1 year [19]. Hip fracture resulting from osteoporosis exhibits high incidence and mortality rates and increases economic burdens worldwide [20]. Patients with osteoporosis are highly likely to experience hip, vertebral, and wrist fractures. Furthermore, previous studies have indicated that osteoporosis can cause alveolar bone loss, which can cause teeth to loosen; hence, patients with osteoporosis tend to have fewer teeth [21–23]. In recent years, dental implants have increasingly been used to treat tooth loss. However, dental implant surgery may fail, and the osseointegration duration may increase if an implant is inserted into tissues with low bone density [24]. Therefore, jawbone osteoporosis is a critical problem for patients who have had dental implant surgery.

Although dual-energy X-ray absorptiometry is most often used clinically to measure bone mass, it provides only a 2D BMD and does not determine the bone shape. Although computed tomography (CT) and quantitative computed tomography (QCT) can be used to measure a 3D BMD, both CT and QCT cannot accurately measure trabecular bone microarchitecture because of low resolution. In a laboratory, microcomputed tomography (micro-CT) is considered the gold standard for assessing bone mass (bone density and trabecular bone microarchitecture) [25]. Several studies have used micro-CT to measure trabecular bone microarchitecture and cortical bone morphology [26–29]. Moreover, micro-CT has high resolution, is nondestructive, and can construct a 3D image of the bone structure, facilitating bone microstructure examination.

Many animal studies have used ovariectomized rats as a clinical model to simulate the bone loss of menopausal women [30–36]. These studies have reported that after a rat is ovariectomized, its trabecular bones at the distal femoral metaphysis [32, 37–39] and inter-radicular septum of the mandibular first molar loosen [40–42]. Although previous studies have shown that the trabecular bones of femurs and jawbones in ovariectomized rats become loose [12, 30, 39, 42–47], few studies have investigated the trabecular bone microarchitecture of the femoral neck and the cortical bone thickness of the mandible [48]. Clinically, hip fracture most frequently occurs in the femoral neck [49, 50]; moreover, the bone mass of the mandible cortical bone is a crucial factor that affects the survival rate of dental implants [51, 52]. Therefore, in the present study, we used micro-CT to evaluate the effects of ovariectomy in rats on the trabecular bone microarchitecture and cortical bone morphology in the femoral neck and mandible. We hypothesized that the both femur and mandible can cause bone loss after ovariectomy. In addition, a positive correlation might exist between the regions of bone morphological and microarchitectural measurements in the femoral neck and mandible.

## Materials and Methods

### Animal preparation and experimental design

Animal experiments were approved (Permit Number: La-1031191) by the Institutional Animal Care and Use Committee of Taichung Veterans General Hospital. All animal experimental procedures were performed in accordance with the pertinent institutional regulations. Female Wistar rats weighing between 220 and 250 g were purchased from BioLASCO Taiwan Co., Ltd. (Taipei, Taiwan). The rats were housed in a temperature- (25°C) and light-controlled room (12: 12 h light–dark cycle), fed standard rat chow, and administered water ad libitum. In addition, the body weight of each rat was recorded every week. The rats were randomly assigned to the control group (n = 6) and ovariectomized group (n = 6). At 8 weeks of age, rats in the ovariectomized group underwent bilateral ovariectomy under isoflurane anesthesia (induction 4%; maintenance 1.5%). Penicillin was administered immediately postoperatively through intraperitoneal injection. The experiment lasted 12 weeks, after which the animals were anesthetized with 3% isoflurane as an inhalation and killed with an overdose of 200 mg/kg of pentobarbital (ip) (Mebumai, SAD, Copenhagen, Denmark). The femurs and mandibles were excised, cleaned of soft tissues, and wrapped in normal saline-soaked gauze for additional ex vivo micro-CT scans.

### Cortical bone morphology and trabecular bone microarchitecture measurements using ex vivo microcomputed tomography

The excised femurs and mandibles were thawed to room temperature for 3 hours before micro-CT scanning. All femurs and mandibles were scanned parallel to the transverse plane and coronal plane, respectively, by using micro-CT. The ex vivo micro-CT images of each femur and mandible were obtained using a Skyscan 1076 micro-CT device (Skyscan, Aartselaar, Belgium). The scanning parameters were set at a voxel resolution of 18.27 μm, 49 kV, 200 μA, 0.5-mm aluminum filter, 500 ms, and a rotation step of 0.8^o^. Furthermore, 180^o^ tomographic rotation scanning was used for the micro-CT scans. Tomographic image reconstruction through Hamming-filtered back projection was performed using NRecon software (Skyscan, Aartselaar, Belgium).

**Fig 1:**
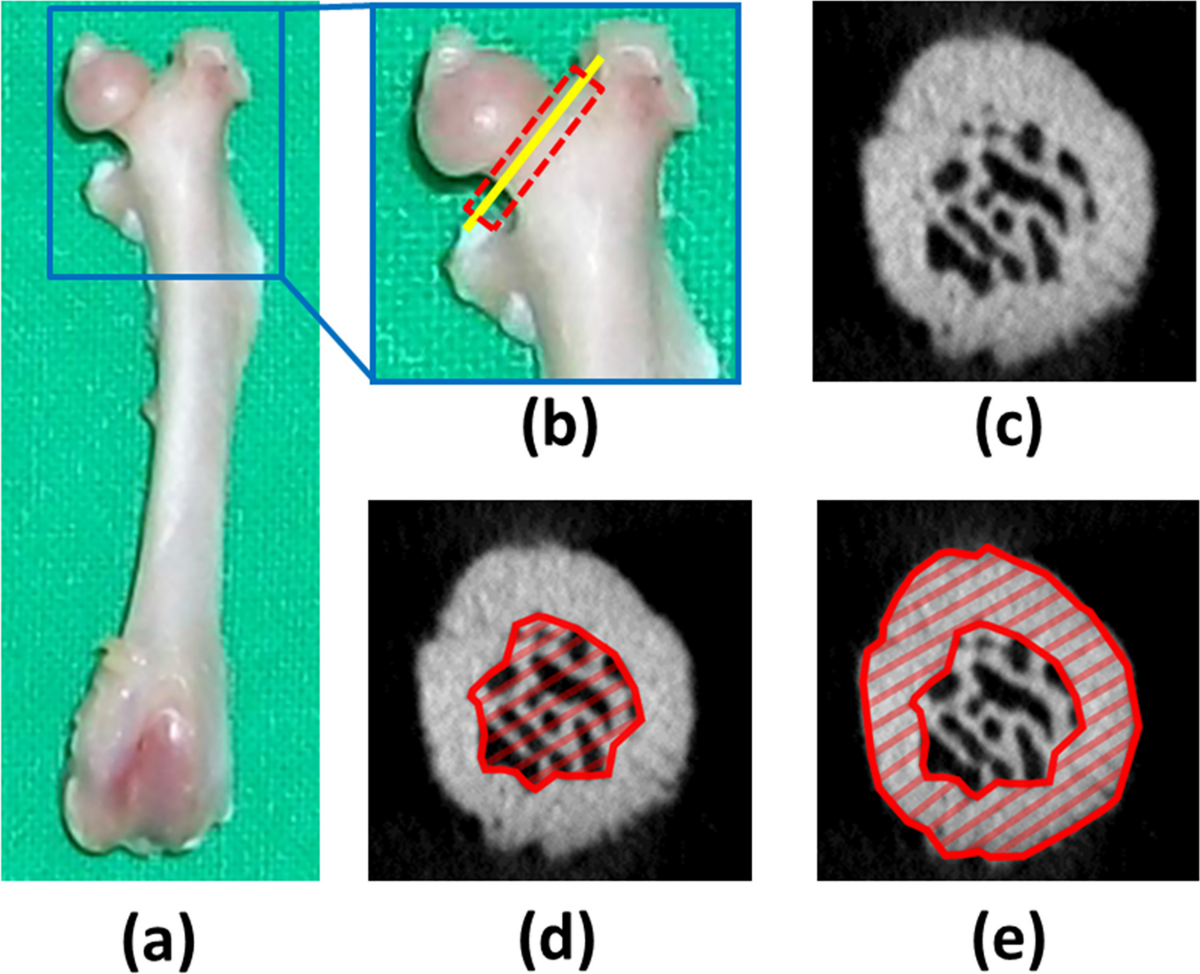
(a) Picture of the left femur. (b) The region of interest (ROI) in the calculated trabecular bone microarchitecture of the femoral neck is indicated by the dotted red rectangle. The ROI in the calculated cortical bone morphology of the femoral neck is indicated by the solid yellow line. (c) Micro-CT image in the yellow line section of the femoral neck. (d) ROI in the trabecular bone microarchitectural measurement. (e) ROI in the cortical bone morphology measurement.

Prior to measurement of the cortical bone morphology and trabecular bone microarchitecture in the femoral necks, the micro-CT images were resliced parallel to the axial plane of the femoral neck. To determine the trabecular bone microarchitecture in the femoral necks, the bone volume fraction (bone volume/total volume, BV/TV, unit = %), trabecular bone thickness (TbTh, unit = mm), trabecular bone separation (TbSp, unit = mm), and trabecular bone number (TbN, unit = 1/mm) [25] were measured using CTAn Skyscan software (Fig 1.). All trabecular bone microarchitectural measurements of the femoral necks excluded the cortical bone. To determine the cortical bone morphology in the femoral necks, single images of the femoral necks were imported into ImageJ software (U.S. National Institutes of Health, Bethesda, MD, USA) to measure the total cross-sectional area (TtAr, unit = mm^2^), cortical area (CtAr, unit = mm^2^), cortical bone area fraction (CtAr/TtAr), and cortical thickness (CtTh, unit = mm) [25].

**Fig 2:**
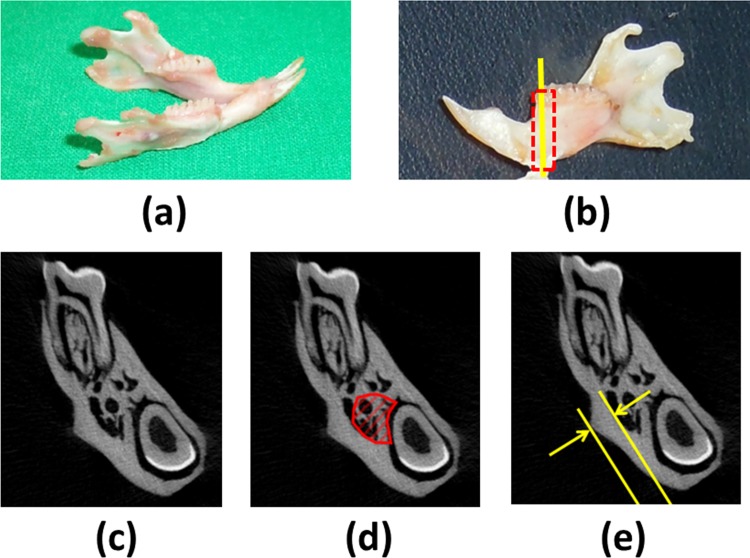
(a) Picture of the right mandible. (b) The region of interest (ROI) in the calculated trabecular bone microarchitecture of the mandible is indicated by the dotted red rectangle. The ROI in the calculated cortical bone morphology of the mandible is indicated by the solid yellow line. (c) Micro-CT image in the yellow line section of the mandible. (d) ROI in the trabecular bone microarchitectural measurement. (e) ROI in the cortical bone morphology measurement.

For the mandible, to determine the trabecular bone microarchitecture in the region below the mandibular first molar, BV/TV, TbTh, TbSp, and TbN [25] were measured using CTAn Skyscan software (Fig 2.). The regions below the first molar were contoured manually in irregular shape, as in previous studies [46, 53]. To determine the cortical bone morphology in the mandible, the length of the masseteric ridge, which is perpendicular to the inner cortical surface (defined as the cortical bone thickness (CtTh)), in the single image of the first molar portion was measured using ImageJ software (Fig 2.).

### Statistical analysis

The cortical bone morphology and trabecular bone microarchitectural parameters of the mandibles and femoral necks in the control and ovariectomized groups were summarized as median ± interquartile ranges. The Mann–Whitney *U* test was used to compare how the cortical bone morphology parameters and trabecular bone microarchitectural parameters of the mandibles and femoral necks differed between the two groups. In addition, the Mann–Whitney *U* test was also used to compare the body weights of the two groups. Spearman analysis was conducted to calculate the correlation coefficients (*r*_*s*_ values) between the femoral neck and mandible values in each group and between the two groups. All statistical analyses were performed using SPSS Version 19 software (IBM Corporation, Armonk, NY, USA). The level of statistical significance was set at *P* < 0.05.

## Results

### Body weight

Body weights of all rats in both groups through the experimental period are listed in S1 Table. The median weights of the two groups are listed in Table 1. Prior to the experiment, no significant difference was observed in weight between the two groups. Comparing both groups showed that except at Weeks 11 and 12, the ovariectomized group was heavier than the control group; for all other time points, no significant difference in weight was recorded between the two groups.

**Table 1:** Body weights of the two groups throughout the experimental period.

Week	Control group				Ovariectomized group				*P*†
	Median	IQR	Max	Min	Median	IQR	Max	Min	
**1**	201.0	12.0	209	187	198.2	9.3	206	189	0.810
**2**	210.5	16.0	237	202	210.5	10.8	223	200	0.470
**3**	230.5	26.0	267	218	249.5	9.0	264	232	0.297
**4**	257.5	27.0	301	239	283.5	22.0	308	266	0.078
**5**	263.5	42.5	318	236	314.5	32.5	330	254	0.150
**6**	277.0	30.5	337	252	305.5	62.8	368	280	0.078
**7**	283.5	22.3	305	248	295.5	36.8	356	255	0.109
**8**	291.0	48.3	364	242	310.0	42.3	359	286	0.297
**9**	309.5	38.0	377	270	328.5	42.0	385	293	0.200
**10**	310.0	39.3	376	279	342.0	35.0	395	325	0.055
**11**	305.0	34.3	331	283	360	34.3	406	340	0.004
**12**	326.5	29.5	395	290	364.0	49.5	409	337	0.037

IQR = interquartile range; Max = maximum; Min = minimum

†Mann–Whitney U-test

### Trabecular bone microarchitecture and cortical bone morphology in the femoral necks

**Fig 3:**
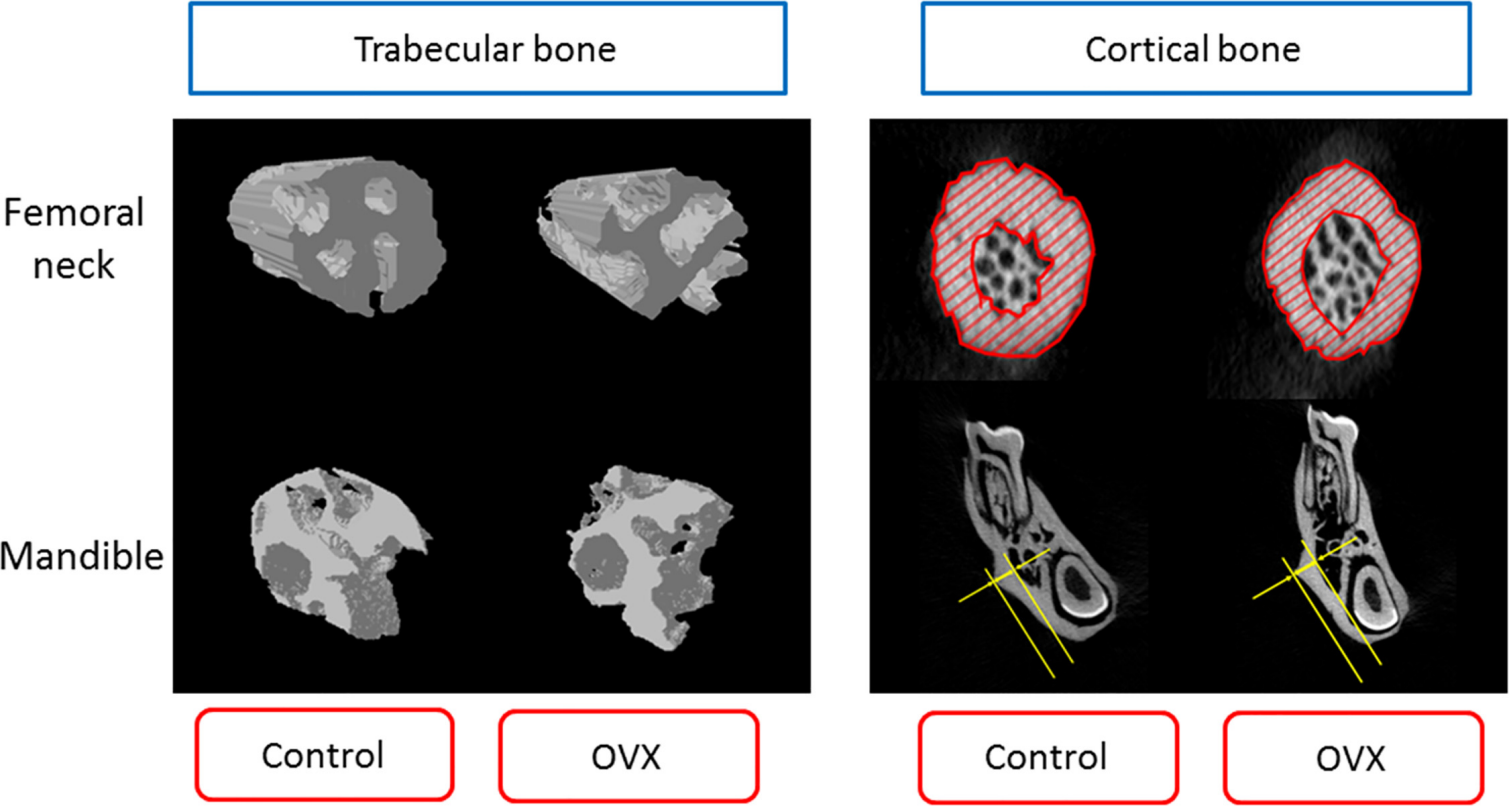
Three-dimensional images of the trabecular bone microarchitecture and cortical bone morphology in a femoral neck and mandible.

The 3D models of the mandibles and femoral necks from rats in the control and ovariectomized groups were constructed using micro-CT (Fig 3). The trabecular bone microarchitectures in both the femoral necks and mandibles of the rats in the ovariectomized group were more loose than those of the rats in the control group. However, there was no significant difference in cortical bone morphology between the control and ovariectomized groups.

Measurement results of all rats in both groups are listed in S2 Table. The measurement results of the trabecular bone microarchitecture and cortical bone morphology in the femoral necks of the control and ovariectomized groups are listed in Table 2. Regarding the trabecular bone microarchitecture in the femoral necks, the results indicated significant differences in BV/TV, TbSp, and TbN between the control and ovariectomized groups (*P* < 0.05). The median BV/TV and TbN of the ovariectomized group were 34.9% and 27.9% lower than those of the control group; the median TbSp of the ovariectomized group was 42.3% higher than that of the control group. The median TbTh of the ovariectomized group was lower than that of the control group; however, there was no significant difference in TbTh between the two groups. Regarding the cortical bone morphology in the femoral necks, there was a significant difference in TtAr between the ovariectomized and control groups; the median TtAr of the ovariectomized group was 10.7% lower than that of the control group. There was no significant difference in CtAr, CtAr/TtAr, or CtTh between the control and ovariectomized groups.

**Table 2:** Morphometric indices corresponding to the trabecular bone microarchitecture and cortical bone morphology in the femoral necks of the control and ovariectomized groups.

**Bone**	**ROI**	**Parameter**	**Unit**	**Control(Median±IQR)**	**Ovariectomized (Median±IQR)**	***P*†**
**Femoral neck**	**Trabecular bone parameter**	BV/TV	%	61.199±11.288	40.329±5.153	0.004*
		TbTh	mm	0.139±0.034	0.125±0.022	0.150
		TbSp	mm	0.130±0.026	0.185±0.066	0.016*
		TbN	1/mm	4.532±0.320	3.268±0.895	0.010*
	**Cortical bone parameter**	TtAr	mm^2^	3.872±1.194	3.456±0.625	0.006*
		CtAr	mm^2^	2.701±0.513	2327±0.156	0.150
		CtAr/TtAr		0.682±0.081	0.659±0.131	0.575
		CtTh	mm	0.494±0.040	0.442±0.083	0.149

BV/TV = bone volume fraction (bone volume/total volume); TbTh = trabecular bone thickness; TbSp = trabecular bone separation; TbN = trabecular bone number; TtAr = total cross-sectional area; CtAr = cortical area; CtAr/TtAr = cortical bone area fraction; CtTh = cortical thickness.

†Mann–Whitney U-test

*Statistical significance (*P* <0.05)

### Trabecular bone microarchitecture and cortical bone morphology in the mandibles

The measurement results of the trabecular bone microarchitecture and cortical bone morphology in the mandibles of the control and ovariectomized groups are listed in Table 3. Regarding the trabecular bone microarchitecture in the mandibles, the results revealed significant differences in BV/TV, TbTh, and TbSp between the two groups (*P* < 0.05). The trabecular bone parameters of the mandibles in the control group (BV/TV = 51.704 ± 6.253% and TbTh = 0.239 ± 0.028 mm) were greater than those in the ovariectomized group (BV/TV = 38.486 ± 9.111% and TbTh = 0.193 ± 0.070 mm). In other words, the BV/TV and TbTh of the ovariectomized rats were respectively 25.56% and 19.24% lower than the BV/TV and TbTh of the healthy rats. In addition, the TbSp of the control group (0.285 ± 0.041 mm) was lower than that of the ovariectomized group (0.322 ± 0.047 mm). In other words, the TbSp of the ovariectomized rats was 12.98% higher than that of the healthy rats. The median TbN of the control group (2.206 ± 0.108 1/mm) was slightly greater than that of the ovariectomized group (2.077 ± 0.429 1/mm); however, there was no significant difference in TbN between the two groups (*P* > 0.05). Regarding the cortical bone morphology in the mandibles, the results indicated no significant difference in CtTh between the control and ovariectomized groups (*P* > 0.05) (CtTh = 0.830 ± 0.107 mm for the control group, and CtTh = 0.832 ± 0.076 mm for the ovariectomized group).

**Table 3:** Morphometric indices corresponding to the trabecular bone microarchitecture and cortical bone morphology in the mandibles of the control and ovariectomized groups.

Bone	ROI	Parameter	Unit	Control(Median±IQR)	ovariectomized (Median±IQR)	*P*†
**Mandible**	**Trabecular bone parameter**	BV/TV	%	51.704±6.253	38.486±9.111	0.004*
		TbTh	mm	0.239±0.028	0.193±0.070	0.037*
		TbSp	mm	0.285±0.041	0.322±0.047	0.025*
		TbN	1/mm	2.206±0.108	2.077±0.429	0.055
	**Cortical bone parameter**	CtTh	mm	0.830±0.107	0.832±0.076	0.837

BV/TV = bone volume fraction (bone volume/total volume); TbTh = trabecular bone thickness; TbSp = trabecular bone separation; TbN = trabecular bone number; CtTh = cortical bone thickness.

†Mann–Whitney U-test

*Statistical significance (*P* <0.05)

### Correlation between mandible and femoral neck

The measurement results of the two regions of interest (ROI) (i.e., the mandible and femoral neck) are listed in Table 4. In both the control and ovariectomized groups, the measurement results of the mandibles were not significantly correlated with the measurement results of the femoral necks (*P* > 0.05). However, simultaneously examining all the rats (whole groups) revealed that the BV/TV of the trabecular bone microarchitecture in the femoral necks was highly correlated with that of the trabecular bone microarchitecture in the mandibles (r_s_ = 0.874, *P* < 0.001). Moreover, the TbSp of the trabecular bone microarchitecture in the femoral necks was highly correlated with that of the trabecular bone microarchitecture in the mandibles (r_s_ = 0.755, *P* = 0.005). Nevertheless, regarding the TbTh and TbN of the trabecular bone microarchitecture and the CtTh of the cortical bone morphology, the measurement results of the mandibles were not significantly correlated with the measurement results of the femoral necks (*P* > 0.05).

**Table 4:** Correlations between the measurements of the regions of interest in the femoral necks and mandibles.

Group	*r_s_*	Trabecular bone microarchitecture				Cortical bone thickness
	p	BV/TV	TbTh	TbSp	TbN	CtTh
**Control group**	***r***_***s***_	0.714	0.371	-0.029	-0.371	0.145
	**p**	0.111	0.468	0.957	0.468	0.784
**Ovariectomized group**	***r***_***s***_	0.257	0.086	0.600	0.086	-0.486
	**p**	0.623	0.872	0.208	0.872	0.329
**Whole groups**	***r***_***s***_	0.874	0.294	0.755	0.322	-0.172
	**p**	<0.001	0.354	0.005	0.308	0.594

## Discussion

Osteoporosis is a critical problem for the health of menopausal women. Osteoporosis reduces bone strength, increases fracture risk, influences quality of life, and even leads to death. Previous studies have shown that after animals were ovariectomized, their systemic BMD correlated with their jaw BMD [12, 30, 42, 47]. However, various assessment methods have been used in numerous studies, and different regions have been measured. The primary reason for the difference in these research findings is that researchers have used different methods for measuring BMD and their ROIs have varied. Few studies have examined the cortical bone thickness of the mandible and the trabecular bone microarchitecture in the femoral neck of ovariectomized rats. This study is the first to investigate the effects of ovariectomy in rats on the trabecular bone microarchitecture and cortical bone morphology in the mandible and femoral neck. The results showed that the trabecular bone microarchitecture in the femoral necks and mandibles of ovariectomized rats became loose; however, the cortical bone morphology in the femurs and mandibles did not change substantially.

Many studies have used ovariectomized rats to investigate osteoporosis resulting from decreased estrogen secretion in menopausal women because the U.S. Food and Drug Administration approved using ovariectomized rats as a clinical model [54]. Therefore, numerous references can be found regarding this topic. Compared with the rat model, previous studies have indicated that rabbits can serve as a superior research model for investigating osteoporosis [45, 55]. Rabbits achieve skeletal maturity at 6 months of age, exhibit more rapid bone turnover than rodents and primates do, and demonstrate significant intracortical remodeling [45, 56]. Thus far, few studies have examined the effect of ovariectomy in rabbits on mandible BMD; hence, in the present study, ovariectomized rats were used instead. Because previous studies have indicated that rats achieve sexual maturity at 6 weeks of age, the rats in the experimental group were ovariectomized in this study at 8 weeks of age.

Currently, micro-CT is considered the gold standard for assessing BMD and trabecular bone microarchitecture [25]. Numerous indicators can be used to describe trabecular bone microarchitecture and cortical bone morphology. Bouxsein et al. asserted that at least four parameters (i.e., BV/TV, TbTh, TbSp, and TbN) must be used in describing trabecular bone microarchitecture and that four parameters (TtAr, CtAr, CtTh, and Cr.Ar/Tt.Ar) must be used in describing cortical bone morphology [25, 28]. Therefore, these indicators were used as the assessment indicators in the present study. However, because the incisors of a rat extend throughout the entire mandible [57] and the mandible contains teeth and mandibular nerves, the TtAr and CtAr of the mandible cannot be measured. Following previous studies [48, 57, 58], we used the cortical bone thickness of the masseteric ridge (CtTh) as an assessment indicator for the cortical bone morphology in the mandible.

Numerous studies have investigated differences in femoral bone quality and quantity between ovariectomized rats and healthy rats [44, 59–63]. Regarding the trabecular bone microarchitecture in the femur, Xiang et al. [59] estimated parameters of the trabecular bone microarchitecture at the distal femoral metaphysis and found significant differences of -73%, -97%, 85%, and -71% in the BV/TV, TbTh, TbSp, and TbN between the ovariectomized and control groups, respectively. Regarding the four trabecular bone microarchitectural parameters of the femoral neck, the results of the present study showed significant differences of -34.1%, -27.9%, and 42.3% in the BV/TV, TbN, and TbSp, respectively, between the ovariectomized and control groups; however, the difference in TbTh was -10.1%, which was not significant. Washimi et al. [44] indicated that regarding the four trabecular bone microarchitectural parameters at the distal femoral metaphysis, significant differences of -58.1%, -13.0%, 200.0%, and -56.9% were observed in the BV/TV, TbTh, TbSp, and TbN between the ovariectomized and control groups, respectively. Giavaresi et al. [61] investigated changes in the trabecular bone microarchitecture at the distal femoral epiphysis in the ovariectomized group and found that compared with the control group, the ovariectomized group showed a significant decrease in BV/TV and TbN of 33% and 21%, respectively, and a significant increase in TbSp of 61%. Regarding the trabecular bone microarchitecture in the femurs of the ovariectomized and control groups, the measurement values obtained in the present study differed from those obtained in previous studies. The reasons may be that the ages and species of the rats used and ovariectomized in this study varied from those in previous studies; most importantly, the ROIs measured using micro-CT in this study varied from those in previous studies. In previous studies, trabecular bone microarchitecture at the distal femoral metaphysis or the distal femoral epiphysis was the ROI [44, 59–63]. Because patients with osteoporosis who undergo a fall commonly sustain hip fracture at the femoral neck [49], this anatomical region was selected as the ROI in the present study. Although the measurement values obtained in this study differed from those obtained in previous studies, the trends observed in this study were consistent with those of previous studies: The trabecular bone microarchitectures in the ovariectomized group were looser than those in the control group.

Except for TbTh, the parametric estimates for BV/TV, TbSp, and TbN in this study indicated that the trabecular bone microarchitecture in the femoral necks of the ovariectomized rats became loose. In contrast to previous studies, this study observed no significant difference in TbTh between the ovariectomized and control groups. Furthermore, compared with the aforementioned studies that have measured the distal femoral metaphysis and distal femoral epiphysis [32, 37–39, 44, 59–63], the present study showed smaller differences between the ovariectomized and control groups. This might be attributable to how the distal femoral metaphysis and distal femoral epiphysis have more trabecular structures than the femoral neck does. Considering the cortical bone morphology parameters of the femoral neck, only the TtAr substantially decreased. The reason may be that after the rats were ovariectomized, decreased estrogen secretion caused bone loss; consequently, cancellous bone that formed from the trabecular bone changed more rapidly than the cortical bone did. In addition, the survival period for the ovariectomized rats was 12 weeks, which is relatively short; hence, the cortical bone did not change substantially. Few studies related to cortical bone morphology have been conducted; thus, no comparison was made between previous studies and this study. Future studies should determine how much time is necessary to produce a substantial change in the cortical bone morphology of ovariectomized rats.

Among studies on the trabecular bone microarchitecture in the mandibles of ovariectomized rats, Irie et al. [42] used micro-CT to determine that compared with healthy rats, the BV/TV and TbTh of the mandibles in the ovariectomized rats decreased by 15% and 16%, respectively, and the TbSp increased by 18%. In the present study, the BV/TV of the ovariectomized rats decreased by 25.56%, markedly greater than the results of Irie et al. Conversely, Tanaka et al. [47] investigated long-term changes in the trabecular bone microarchitecture in the mandibles of ovariectomized rats and found that compared with healthy rats, the BV/TV, TbTh, and TbN of the ovariectomized rats decreased by 75%, 46%, and 58%, respectively, and the TbSp increased by 354%, greater than the results of the present study. No significant difference in TbN between the healthy and ovariectomized rats was observed; however, compared with the healthy rats, the TbSp of the ovariectomized rats increased by 13.0%, substantially different from the results of this study. The main reason for this difference was that in previous studies, the ROI was mostly the inter-radicular septum, which is a small region and close to the tooth root. The tooth root is surrounded by periodontal ligament (PDL), and different biting forces squeeze the PDL to various extents, thereby producing different bone remodeling rates. Following previous studies [46, 53, 57], we measured the region below the first mandibular molar and found no significant difference in TbN between the ovariectomized and control groups, whereas the BV/TV, TbTh, and TbSp results indicated that the region was loose. Compared with healthy rats, the decreases in BV/TV of the mandibles and femoral necks of the ovariectomized rats were 25.56% and 34.1%, respectively. This indicated that although the femoral necks were more sensitive to ovariectomy than the mandibles, the mandibles of the ovariectomized rats still became loose. Therefore, the mandibles of menopausal women may exhibit a decrease in bone mass, and dental implant surgery for such women should be performed with caution [51, 52]. The results of the cortical bone morphology measurements of both the femoral necks and mandibles revealed no significant difference between the ovariectomized and healthy rats. The cortical bone morphology did not change substantially, possibly because the period following the ovariectomies was short [30].

We analyzed the correlation between the measurement results of the mandibles and femoral necks and found that the BV/TV and TbSp of the trabecular bone microarchitecture in the femoral necks were highly correlated with those of the trabecular bone microarchitecture in the mandible (r_s_ = 0.874 for BV/TV, *P* < 0.001; r_s_ = 0.755 for TbSp, *P* = 0.005). However, the CtTh of the cortical bone morphology in the femoral necks was not correlated with that of the cortical bone morphology in the mandibles; the reason may be that osteoporosis of the cortical bone, resulting from ovariectomy, occurred slowly. Few studies have explored the correlation between the osteoporosis conditions of the cortical bone in femurs and mandibles of ovariectomized animals. In future studies, the survival period of ovariectomized rats could be increased to further explore issues related to osteoporosis. Previous studies have indicated that after rats were ovariectomized, osteoporosis of the trabecular bone microarchitecture in their jawbones was correlated with osteoporosis of the trabecular bone microarchitecture in their other bones [12, 30, 42, 47]. The present study further confirmed this observation, despite targeting the region below the first mandibular molar and the region of the femoral neck as the ROIs.

This study had several limitations. Specifically, whether the results obtained from the osteoporosis animal experiment of this study can be applied to the human body must be verified. Because of its limited scan range, micro-CT cannot be applied to the human body. In the future, if the resolution of dental cone beam computed tomography improves, the method can be adopted to measure the trabecular bone microarchitecture in the jawbone. Furthermore, we measured only the trabecular bone microarchitecture and cortical bone morphology in the mandibles and femoral necks but did not explore osteoporosis of the spine or wrist. Based on the sample size, the measurements were analyzed by conducting nonparametric tests. More specimens or a power test should be performed before experiments in the future. In this study, to ensure high image quality, the femurs and mandibles of all rats were scanned using ex vivo micro-CT. However, in vivo scanning would enable monitoring the bone microstructural changes in a longitudinal study. Finally, we mainly used micro-CT to analyze bone mass but did not perform a biomechanical test to measure bone strength. If these limitations can be overcome in the future, then osteoporosis of the femur and jawbone in menopausal women can be further understood.

## Conclusion

Based on the experimental setup and limitations, this study’s conclusions regarding osteoporosis in ovariectomized rats are as follows:

1. The trabecular bone microarchitecture in the mandibles and femoral necks of the ovariectomized group were significantly looser than those of the control group; this result can be observed in particular from two parameters (i.e., BV/TV and TbSp).
2. Regarding the cortical bone morphology in the mandibles and femoral necks, the TtAr of the femoral necks of the ovariectomized group was significantly smaller than that of the femoral necks of the control group; there was no significant difference in the remaining parameters between the two groups.
3. The trabecular bone microarchitectural parameters (particularly, the BV/TV and TbSp) of the femoral necks and mandibles were highly correlated.

## Supporting Information

S1 Table Body weights of all rats in both groups through the experimental period. (PDF)

S2 Table Measurement results of all rats in both groups. (PDF)
