# Supplementary material for: Cortical Bone Morphological and Trabecular Bone Microarchitectural Changes in the Mandible and Femoral Neck of Ovariectomized Rats
Source: PLoS One. 2016 Apr 29;11(4):e0154367. doi: 10.1371/journal.pone.0154367 (PMC4851407; doi:10.1371/journal.pone.0154367)
Supplement: S1 Table — (PDF) [file pone.0154367.s001.pdf]

S1 Table. Body weights of all rats in both groups through the experimental period.

		Week											
Group		1	2	3	4	5	6	7	8	9	10	11	12
Control	1	187	210	228	253	275	278	286	302	321	324	323	327
	2	192	202	218	239	252	300	305	316	322	329	331	331
	3	206	230	254	279	299	337	286	364	377	376	292	395
	4	209	237	267	301	318	252	258	242	279	296	286	290
	5	198	209	221	246	236	260	248	259	270	279	283	292
	6	204	211	233	262	250	276	281	280	298	286	318	326
	Median*	201	210.5	230.5	257.5	263.5	277	283.5	291	309.5	310	305	326.5
	IQR	12	16	26	27	42.5	30.5	22.25	48.25	38	39.25	34.25	29.5
	Max	209	237	267	301	318	337	305	364	377	376	331	395
	Min	187	202	218	239	236	252	248	242	270	279	283	290
OVX	1	206	214	251	283	314	354	356	359	385	395	406	409
	2	195	202	232	266	274	280	297	308	317	325	350	337
	3	195	207	248	284	315	326	339	357	372	375	381	390
	4	205	223	264	308	330	285	255	286	327	335	342	343
	5	189	200	243	270	254	368	291	312	293	333	340	338
	6	202	214	254	299	317	284	294	302	330	349	370	385
	Median*	198.5	210.5	249.5	283.5	314.5	305.5	295.5	310	328.5	342	360	364
	IQR	9.25	10.75	9	22	32.5	62.75	36.75	42.25	42	35	34.25	49.5
	Max	206	223	264	308	330	368	356	359	385	395	406	409
	Min	189	200	232	266	254	280	255	286	293	325	340	337
