# Supplementary material for: Cortical Bone Morphological and Trabecular Bone Microarchitectural Changes in the Mandible and Femoral Neck of Ovariectomized Rats
Source: PLoS One. 2016 Apr 29;11(4):e0154367. doi: 10.1371/journal.pone.0154367 (PMC4851407; doi:10.1371/journal.pone.0154367)
Supplement: S2 Table — (PDF) [file pone.0154367.s002.pdf]

S2 Table. Measurement results of all rats in both groups.

		Mandibule						Femoral neck							
		Trabecular bone				Cortical bone		Trabecular bone				Cortical bone			
Group	No.	BV/TV (%)	Tb. Th (mm)	Tb. Sp (mm)	Tb. N (1/mm)	CtTh (mm)		BV/TV (%)	Tb. Th (mm)	Tb. Sp (mm)	Tb. N (1/mm)	CtAr (mm2)	TtAr (mm2)	CtAr/TtAr	CtTh(mm)
Control	1	54.580	0.253	0.272	2.157	0.834		62.762	0.147	0.143	4.275	3.151	4.826	0.653	0.484
	2	51.522	0.236	0.261	2.187	1.011		54.068	0.119	0.134	4.538	2.606	3.853	0.676	0.488
	3	59.420	0.267	0.291	2.226	0.887		76.934	0.166	0.112	4.627	2.730	3.776	0.723	0.568
	4	49.154	0.219	0.333	2.240	0.787		59.636	0.132	0.158	4.527	2.417	3.287	0.735	0.500
	5	49.665	0.242	0.302	2.055	0.819		58.744	0.121	0.124	4.863	3.046	4.913	0.620	0.455
	6	51.885	0.232	0.279	2.241	0.826		66.172	0.151	0.125	4.396	2.671	3.891	0.687	0.500
	Median=	51.704	0.239	0.285	2.206	0.830		61.199	0.139	0.130	4.532	2.701	3.872	0.682	0.494
	IQR=	6.253	0.028	0.041	0.108	0.107		11.288	0.034	0.026	0.320	0.513	1.194	0.081	0.040
	Max=	59.420	0.267	0.333	2.241	1.011		76.934	0.166	0.158	4.863	3.151	4.913	0.735	0.568
	Min=	49.154	0.219	0.261	2.055	0.787		54.068	0.119	0.112	4.275	2.417	3.287	0.620	0.455
OVX	1	30.052	0.146	0.336	2.063	0.827		41.599	0.122	0.183	3.457	2.318	3.520	0.665	0.456
	2	34.473	0.160	0.324	2.154	0.874		39.410	0.128	0.186	3.080	2.459	3.391	0.725	0.494
	3	42.277	0.202	0.321	2.092	0.777		40.828	0.137	0.223	2.989	2.236	2.965	0.754	0.527
	4	43.084	0.246	0.305	1.751	0.837		50.986	0.115	0.140	4.446	2.201	3.371	0.653	0.429
	5	40.065	0.183	0.317	2.188	0.815		36.941	0.099	0.154	3.739	2.357	4.023	0.586	0.424
	6	36.907	0.220	0.436	1.679	0.904		39.829	0.131	0.215	3.032	2.336	3.852	0.606	0.406
	Median=	38.486	0.193	0.322	2.077	0.832		40.329	0.125	0.185	3.268	2.327	3.456	0.659	0.442
	IQR=	9.111	0.070	0.047	0.429	0.076		5.153	0.022	0.066	0.895	0.156	0.625	0.131	0.083
	Max=	43.084	0.246	0.436	2.188	0.904		50.986	0.137	0.223	4.446	2.459	4.023	0.754	0.527
	Min=	30.052	0.146	0.305	1.679	0.777		36.941	0.099	0.140	2.989	2.201	2.965	0.586	0.406
